# Physician-modified funnel-shaped covered stent for selective false lumen exclusion in chronic type B aortic dissection

**DOI:** 10.1177/17085381241289811

**Published:** 2024-10-04

**Authors:** Lorenzo Torri, Petroula Nana, Giuseppe Panuccio, José Ignacio Torrealba, Daour Yousef el Sarhan, Tilo Kölbel

**Affiliations:** German Aortic Center, Department of Vascular Medicine, 37734 University Medical Center Eppendorf, Hamburg, Germany

**Keywords:** Physician-modified, balloon-expandable covered stent, false lumen occlusion, type B aortic dissection, aorta

## Abstract

**Purpose:**

To describe the technique of off-centering a balloon-expandable covered stent for selective occlusion of a distal entry tear (ET) in a patient, conservatively treated for chronic type B aortic dissection (cTBAD), presenting FL expansion.

**Technique:**

A 63-year-old male, with conservatively managed cTBAD, presented at follow-up with FL partial thrombosis and expansion (thoracic aorta FL from 21 mm to 27 mm and abdominal aorta FL from 11 mm to 15 mm in 6 months). No proximal ET was identifiable. Distal FL perfusion was caused by an ET in the abdominal aorta feeding a 2 mm accessory renal artery (ARA). As the aortic diameter was below the threshold for endovascular repair, a selective occlusion of the distal ET and ARA was planned. A balloon-expandable covered stent was modified by off-centering the covered stent proximally and resulting in a funnel-shape occluder after deployment across the ET into the ARA. To prevent type Ic endoleak due to possible FL expansion caused by an intra-operatively detected phrenic artery (PA), coils were deployed into the lumen of the modified stent and the ARA. The pre-discharge computed tomography angiography showed exclusion of both the ARA and ET and a type 2 endoleak from the PA.

**Conclusion:**

A balloon-expandable covered stent can be modified by off-centering the covered stent resulting in a funnel shape to adapt to different diameter requirements.

## Introduction

Thoracic endovascular aortic repair (TEVAR) has been increasingly used in the treatment of chronic type B aortic dissection (TBAD) to occlude primary entry tears and prevent false lumen (FL) expansion.^1^ TEVAR in subacute TBAD enhances aortic remodeling.^2,3^ However, almost 30% of patients still present aortic dilation, especially to the abdominal aortic segment, due to distal entry tears or large diameter collaterals arising from the FL.^2,3^ Complete FL thrombosis after TEVAR has been related to positive aortic remodeling,^4,5^ while in about 1/3 of patients the FL continues to expand due to distal false lumen perfusion.^6,7^ Strategies focusing on endovascular FL exclusion in chronic TBAD have shown encouraging outcomes.^6,8–11^ The continuous evolution of techniques and materials highlights the importance of FL management in TBAD, while the great variability of dissection morphology and aortic anatomy requiring individualized treatment strategies.^6,8–11^

Herein, we report the use of an off-centered balloon-expandable covered stent for selective occlusion of a distal entry tear, in direct communication with an accessory renal artery (ARA), in a patient with chronic TBAD and FL expansion.

A written consent has been assigned by the patient, confirming his agreement to publication of the case details and images.

## Technique

### Case presentation

**Figure 1.**
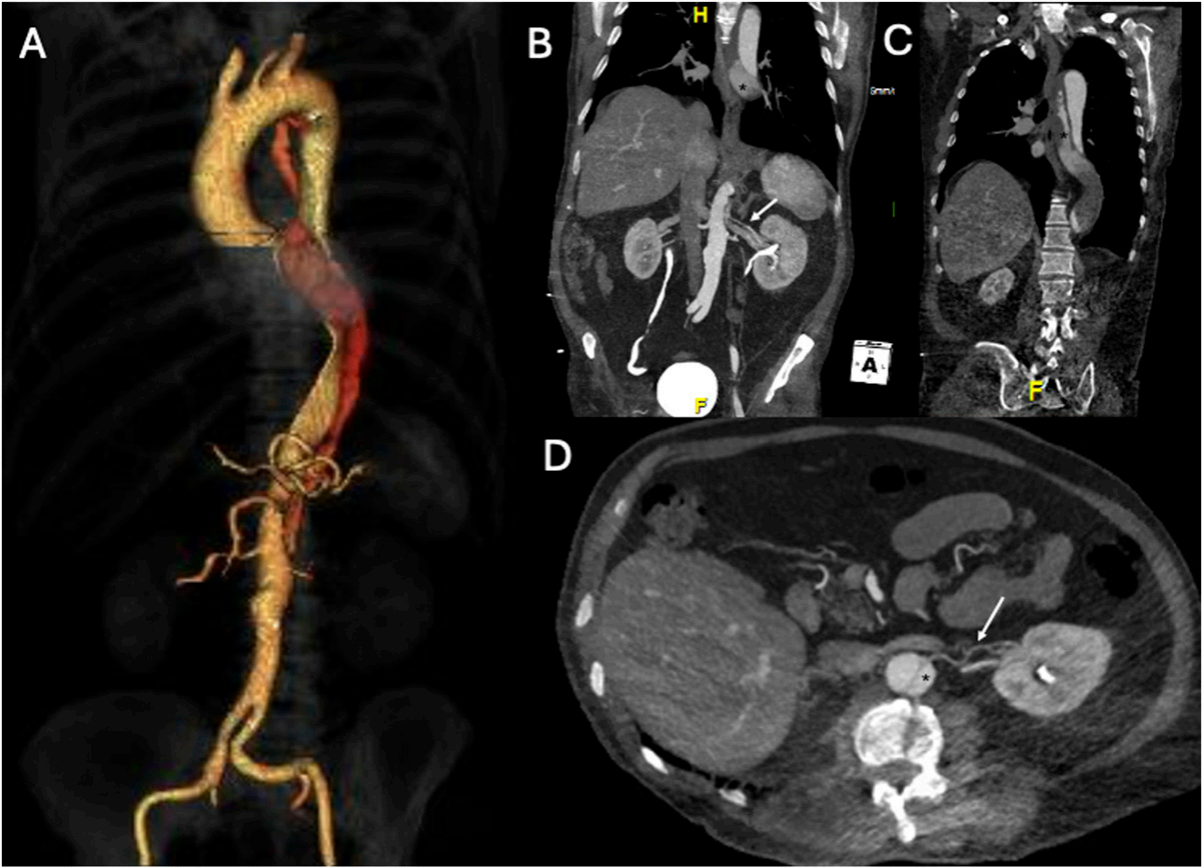
April 2022 pre-operative three dimensions volume rendering (3D VR) and Multi-Planar Reconstruction (MPR) Computed Tomography Angiography (CTA) of Type B aortic dissection (TBAD); (A) 3D VR of total aorta; (B-C-D) MPR CTA showing both false lumen (FL, star) and left accessory renal artery (ARA, arrow) in three different images: (B) MPR in an oblique coronal plane showing the entry tear (ET) in the abdominal aorta (zone 8) in direct communication with left ARA (arrow) and the FL perfusion at this level (star), (C) MPR in an oblique coronal plane showing FL patency up to zone 4 (star), (D) MPR in an oblique axial plane showing the left ARA (arrow) in direct communication with the ET.

A 73-year-old male patient presented with an uncomplicated chronic TBAD (B_3,10_ as SVS-STS reporting standard), incidentally found in a computed tomography scan in April 2022 (Figure 1(A)), performed for suspected pulmonary embolism due to interruption of anticoagulant therapy after a traumatic leg injury. He had a history of hypertension and atrial fibrillation under anticoagulant therapy (Apixaban, 5 mg twice daily). No symptoms or signs that could be related to the acute incident of aortic dissection were identified. The TBAD extended distally to the common iliac arteries, with an entry tear located in the abdominal aorta, (SVS-SVT zone 8; (Figure 1(B)–(D)), in direct communication with a 2 mm left accessory renal artery (ARA), preserving FL patency up to aortic zone 4 (Figure 1(B), (C)). Conservative treatment was decided, and the patient remained under clinical and imaging surveillance.

**Figure 2.**
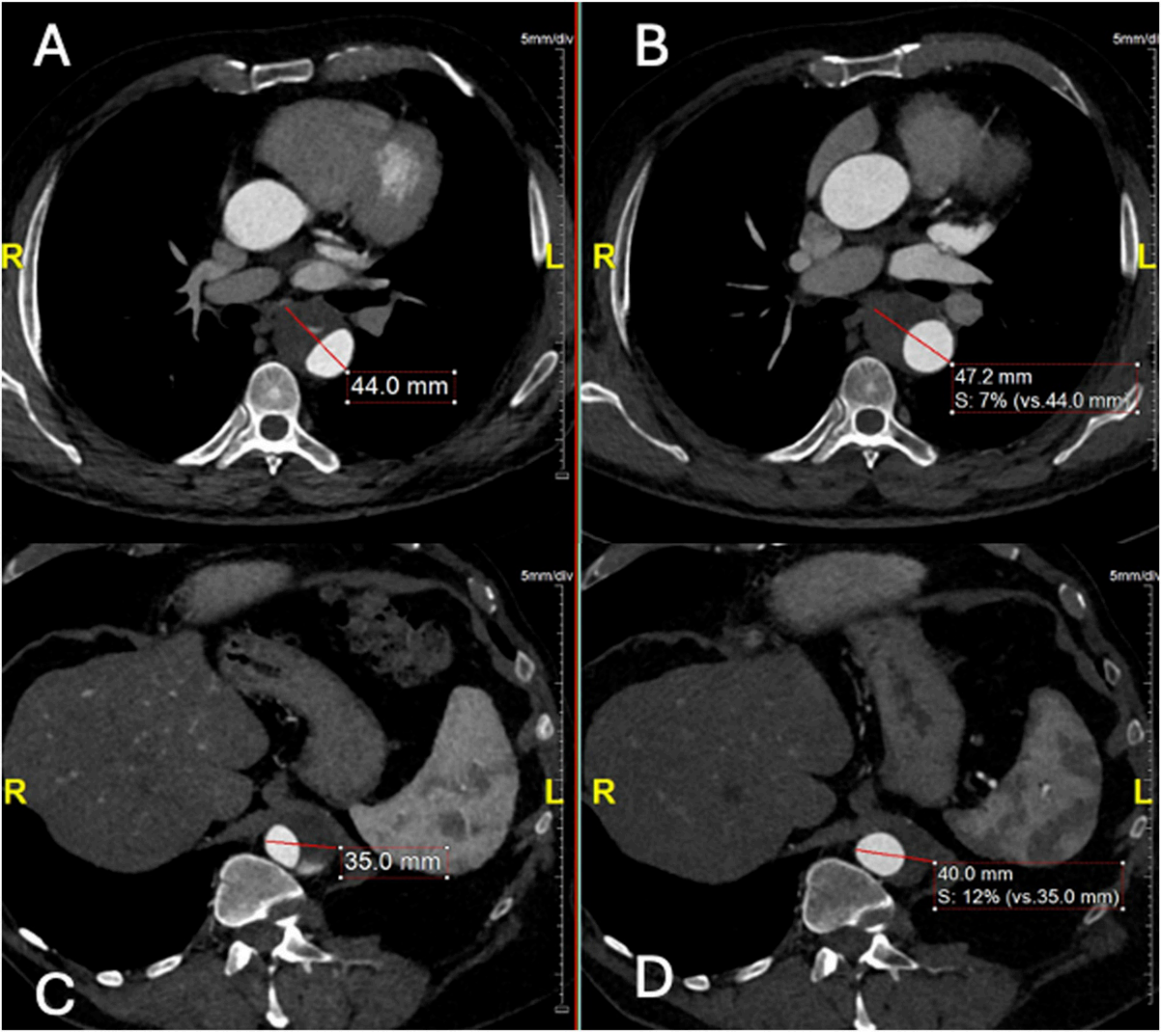
Aortic diameters comparison at 18 months follow-up. Axial CTA demonstrating (A) the diameter of thoracic aorta (44 mm) in April 2022, (B) the diameter of thoracic aorta (47.2 mm) in September 2023, (C) the diameter of abdominal aorta in April 2022 (35 mm), (D) the diameter of abdominal aorta (40 mm) in September 2023.

In the computed tomography angiography (CTA) after 1 and 3 months, no significant aortic alterations were detected. The 6-month CTA showed complete FL thrombosis in zone 4, without significant differences regarding diameters and FL perfusion restricted to zone 8. The CTA after 18 months showed no modifications in FL perfusion in zone 8 but with FL expansion (from 21 mm to 27 mm in the thoracic aorta and 11 mm to 15 mm in the abdominal aorta). An increasing total aortic diameter was also recorded, in both the thoracic (from 44 mm to 47 mm) and abdominal segment (35 mm to 40 mm, Figure 2), which was attributed to TL and FL expansion. This FL expansion under best medical therapy did not fulfill classical indication thresholds for endovascular repair, which in that case would require not only TEVAR but also thoracoabdominal extension with a fenestrated/branched repair. As there was a clear growth tendency of the thoracoabdominal aorta (6 mm FL expansion in thoracic aorta, 4 mm FL expansion in abdominal aorta, 3 mm diameter growth in thoracic aorta, 5 mm diameter growth in abdominal aorta in 6 months), we recommended a less invasive approach already at this early stage in order to occlude the single detectable entry tear in connection with the ARA.

### Procedure

**Figure 3.**
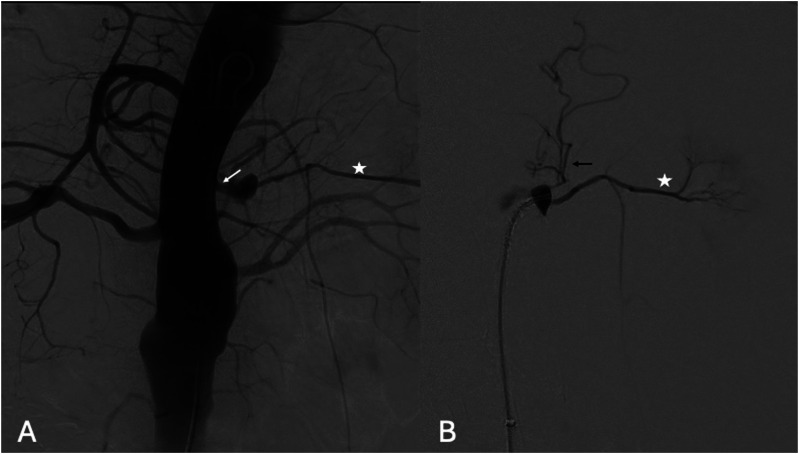
Digital subtraction angiography (DSA) showing (A) true lumen aortography with the ET (white arrow) in direct communication with left ARA (star), (B) selective false lumen DSA with the left ARA (star) and a small phrenic artery (black arrow).

The procedure was performed in a hybrid operating room with a fixed imaging system, under fusion guidance. The patient was in supine position under general anesthesia. A single percutaneous left common femoral artery (CFA) access, using a 6Fr × 55 mm sheath (Flexor, Cook Medical Bloomington, IN, USA), was performed under ultrasonographic guidance. Systemic heparinization at 100 IU/kg with a target activated clotting time >250 s was obtained. The initial angiography localized the entry tear and left ARA (Figure 3). Using a hydrophilic .035’ guidewire (Terumo Medical Corporation, NJ, USA) and a 90 cm 5F Bernstein (Cordis Corporation, Florida, USA) catheter, the FL was catheterized, and the hydrophilic wire was exchanged to a 0.035’ Rosen wire (Cook Medical, Bloomington, IN, USA).

**Figure 4.**
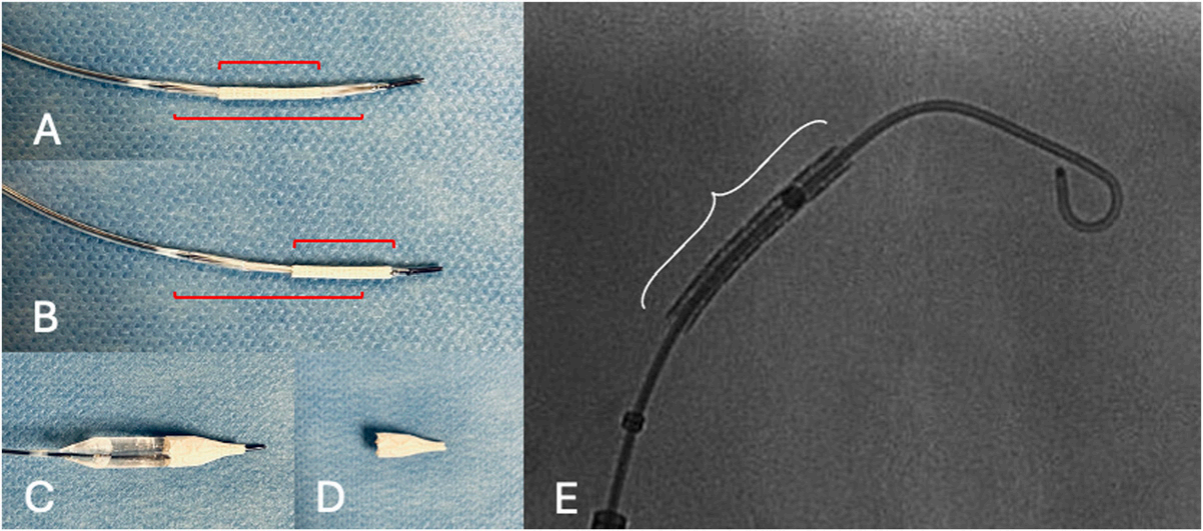
On-table modification. (A) normal balloon and stent position (B) Stent moved afterwards on the balloon, in A and B the red lines show the stent position in relation to the balloon markers, (C-D) Image for demonstration purpose of the stent during ballooning (C) and after (D) with a «funnel shape», (E) Intraoperative fluoroscopy of the unmounted stent, white curly bracket shows the stent position in relation to the balloon markers.

**Figure 5.**
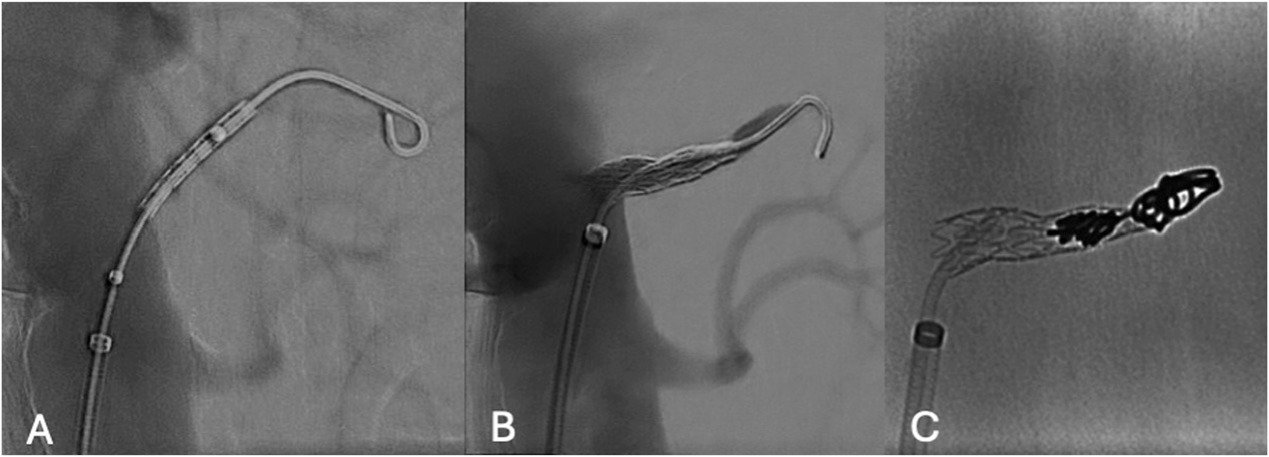
(A) Initial pre-deployment DSA, (B) after deployment stent positioning, and (C) in stent coil fluoroscopy.

A balloon-expandable covered stent (5 × 22 mm, Advanta V12, Atrium Medical Corporation, NH) was modified on a side-table, by off-centering the covered stent over the balloon (advancing the covered stent to the tip of the balloon), in order to create a funnel shape after deployment (Figure 4). The modification procedure was performed simply by pulling the stent towards the tip of the balloon with the right hand, while holding the shaft steady with the left hand. The distal part of the stent would be positioned into the ARA while the proximal part would be used to occlude the ET. After deployment, flaring with a 7 × 20 mm balloon (Armada, Abbott, Lake Forest, IL, USA) was performed to enhance seal in the ET (Figure 5(A), and (B)). As we detected intra-operatively that the FL was not only connected to the ARA but also to a small phrenic artery we decided to occlude the ARA and the covered stent by coil embolization (Nester 4 mm, Cook Medical, Bloomington, IN, USA) in the covered stent and the ARA to prevent type Ic endoleak (Figure 5(C)), due to possible FL expansion caused by the phrenic artery, expecting complete thrombosis of the ARA. Completion angiography confirmed the exclusion of the FL. Sheaths and wires were retrieved, and manual compression was applied over the left CFA. The duration of the procedure was 40 min, with a total fluoroscopy time of 10:37 min and radiation dose at 34.7 Gy.cm^2^.

**Figure 6.**
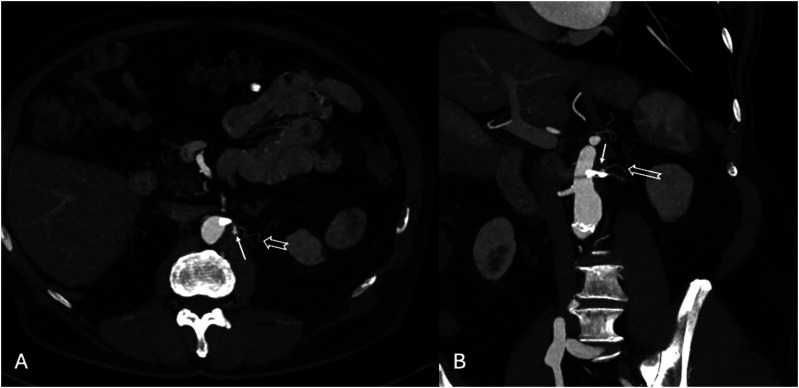
Pre-discharge CTA showing type II endoleak (white arrow) from a 1 mm phrenic artery (empty arrow). (A) Maximum intensity projection (MIP) axial CTA (B) MIP MPR CTA in an obliqual coronal plane.

**Figure 7.**
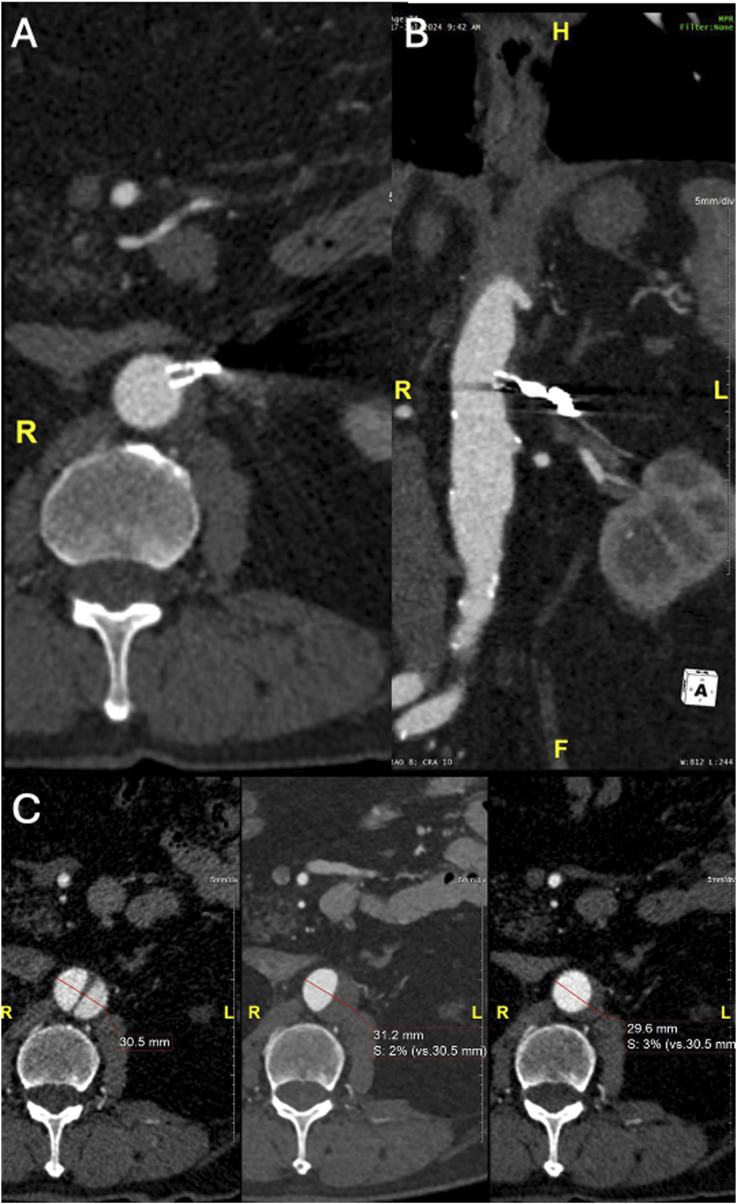
Six-month Follow-up CTA showing complete FL thrombosis and comparative aortic diameter of pre–post procedure and FU CTA. (A) Axial CTA (B) MPR CTA in an obliqual coronal plane (C) Comparative aortic diameter from right to left of pre–post procedure and FU CTA at the level of the distal tear.

Post-operatively, the patient was transferred directly to the ward. The pre-discharge CTA confirmed appropriate position of the stent, with occlusion of the ARA and entry tear. A type II endoleak from the small phrenic artery was detected (Figure 6). The post-operative course was uneventful, and the patient was discharged the 2nd post-operative day. The 6-month follow-up CTA showed complete thrombosis of the FL in zone 8 (zone 4 was already thrombosed) with no expansion or increase in diameter of the FL. Additionally, the phrenic artery was no longer visible (Figure 7).

## Discussion

The fate of the FL plays a crucial role for the clinical success of endovascular repair in aortic dissection cases.^6^ Distal re-entry tears in the descending thoracic or thoracoabdominal aorta permit retrograde flow to the FL and affect aortic remodeling and further, patients’ long-term survival.^2,3,5,6^ High false lumen expansion rates despite proximal entry-tear coverage by TEVAR highlight the necessity for complete FL exclusion to ensure the durability of endovascular aortic dissection repair.^2,3^

According to the current guidelines, the patient was initially managed with conservative treatment.^5^ Diameters did not indicate the need for a complete aortic exclusion at that time, which is safe but associated with 15% morbidity and almost 50% reintervention rates at 24 months.^4,5,12^ FL expansion though led the decision for entry tear and ARA exclusion. Despite its limited diameter at 2 mm, the ARA seemed to affect FL hemodynamics and preserved patency.

Endovascular repair with aortic endografts, aims to seal the main entry tears, in order to achieve cessation of flow and depressurization of the FL.^6,13^ Nevertheless, it falls short of achieving total FL thrombosis and additional strategies focusing on FL occlusion and reinterventions are frequently needed.^4,5,12^ False Lumen Endografts (FLE) also known as Candy-Plug, as well as other embolic material, including coils, glue and plugs have been used and showed encouraging outcomes in terms of total FL thrombosis and aortic remodeling.^6,9–11,14,15^ However, not all patients conform to the aforementioned techniques. The presence of a small distal entry tear and the lack of endovascular management of the true lumen hampered the application of FLE while the exclusive use of coils and/or plugs would not permit the concomitant selective embolization of the ARA and entry tear. The use of a plug or coils within the ARA would have selectively closed the artery but not the ET, so the FL could have been fed by the ET. If instead, we had used a plug to close the ET, there would have been a risk that the FL could have been perfused retrogradely from the ARA. The use of a candy-plug in this case would require not only TEVAR but also thoracoabdominal extension with a fenestrated/branched repair and diameters to not allow this kind of repair.

Despite technical success with distal entry tear exclusion at completion angiography, the pre-discharge CTA revealed a new type II EL, from a small phrenic artery in the current case. Alterations in FL hemodynamics affect collateral arteries’ flow and may lead to unpredictable behavior and endoleak formation.^13,14^ In this case, the small phrenic artery was not detected on pre-operative CTA and became visible first during treatment. As often happens, the small phrenic artery thrombosed over the following 6 months, leading to the subsequent thrombosis of the FL.

The use of an off-centered balloon-expandable covered stent allowed to combine placement into a small ARA, which otherwise may have been ruptured by the 5 mm covered stent. Other techniques, like coil embolization of both, the ARA and phrenic artery or placement of a septal occlude could have been used as alternatives in this specific case, but we believe that the described physician-modified technique offers advantages, in terms of the ease of use and limited catheterization efforts. Coil embolization of the FL and its side branches, as well as the use of plugs for entry tear occlusion has been used with suboptimal results according to the published limited experience, mainly attributed to the need of reintervention.^15^ It may become a useful adjunctive technique in specific anatomies or can be used in f/bEVAR to save small vessel (<3 mm diameter) that are normally sacrificed. However, it should be acknowledged the potential risk of stent dislodgment, especially after on-table modifications. To avoid such a technical pitfall, crimping the stent onto the balloon should be performed with caution, and possibly be assisted using fabric ties. Since dislodgment often occurs during the initial introduction steps, adjustment of the introducer’s valve pressure, if possible, could be used as an additional measure. Continuous follow-up is of major importance in patients with TBAD, regardless the initial treatment, due to uncertain aortic evolution and need of reintervention.

## Conclusion

A balloon-expandable covered stent can be modified by off-centering the covered stent resulting in a funnel shape to adapt to different diameter requirements.
